# A retrospective study of mortality in Eurasian lynx (*Lynx lynx*) in UK zoos

**DOI:** 10.1002/zoo.21476

**Published:** 2019-01-22

**Authors:** Joseph Heaver, Michael Waters

**Affiliations:** ^1^ Department of Pathobiology and Population Sciences Royal Veterinary College London UK

**Keywords:** carcinoma, disease, European, felid, zoological

## Abstract

IUCN currently classifies the Eurasian lynx (*Lynx lynx*) as “Least Concern,” however, across its six to nine subspecies, some isolated populations are classified as “Endangered” or “Critically Endangered.” Despite this and the species’ relative ubiquity in European zoos, a retrospective mortality study of a captive population has not previously been performed. By analyzing necropsy reports, animal records, and the European studbook, we were able to ascertain a cause of death for 38 (73%) of the 52 recorded lynx deaths in UK zoos during the study period (January 1, 2000 to November 1, 2015). “Culling” as part of population management was the most common cause of death (21%) followed by neoplastic (16%), circulatory (11%), neurological (11%), and genitourinary (11%) disease. “Geriatric” individuals accounted for 62% of lynx to die within the study period, 23% were “neonates” and 15% “adults.” Neoplasia, circulatory disease, and culling were the leading causes of death in each of these age categories, respectively. Excluding “culls” and “neonates,” the mean age at death was 18.81 ± 0.42 years, consistent with existing data. Squamous cell carcinoma was reported in three individuals (8%) and suspected idiopathic epilepsy in four individuals (11%), warranting further investigation. Intraspecific killing (3%) and neonatal mortality, excluding culls, (14%) were reported with lower prevalence than expected based on previous studies of similar species. The lack of data available and high incidence of culling of individuals with a high inbreeding coefficient highlights the need for improved record‐keeping and consultation with the studbook coordinator, respectively.

**Abbreviations**
COI: coefficient of inbreeding
EAZA: European Association of Zoos and Aquaria
IUCN: International Union for Conservation of Nature
PME: post‐mortem examination
SCC: squamous cell carcinoma
ZIMS: Zoological Information Management System

## 1 INTRODUCTION

The Eurasian lynx (*Lynx lynx*) is the largest species of its genus, which also includes the Iberian lynx (*Lynx pardinus*), the Canada lynx (*Lynx canadensis*), and the bobcat (*Lynx rufus*).

There are between six and nine subspecies of the Eurasian lynx (Breitenmoser et al., 2015), four of which are currently kept in UK zoos. These are the Northern lynx (*L. l. lynx)*, the Siberian lynx (*L. l. wrangeli*), the Carpathian lynx (*L. l. carpathicus*), and the Turkestan lynx (*L. l. isabellinus*). There are currently 47 (20.27) Eurasian lynx in UK zoos registered on the Zoological Information Management System (ZIMS) database with at least another eight individuals (3.5) at institutions which do not utilize ZIMS. Northern lynx is the most common of the subspecies in UK zoos (*n* = 21; 7.14), followed by the Carpathian lynx (*n* = 14; 8.6). Siberian lynx (*n* = 3; 1.2), Turkestan lynx (*n* = 2; 1.1), and Lynx of unknown subspecies (*n* = 9; 4.5) make up a minority of the captive UK population. Of the estimated 55 captive Eurasian lynx in the UK, 33 (12.21) were registered in the European studbook as of December 31, 2014.

The species’ native range once spread over the majority of Europe, absent only on the Iberian Peninsula (Kratochvil, 1968), where the Iberian lynx occurs. However, in the mid‐twentieth century the global population of Eurasian lynx became perilously low and the species became extinct in continental Western Europe (Kratochvil, 1968). This decline was largely a result of poaching, deforestation and reduced populations of prey animals (Breitenmoser, 1998). However, by the end of the 20th century the species’ population had risen to levels thought to be the highest in centuries following reintroduction efforts aided by improved international legislation enforcing the protection of both the Eurasian lynx and the prey species upon which the felid relies (Hayward and Somers, 2009).

The International Union for Conservation of Nature (IUCN) classify the species as “least concern” with a “stable” population (Breitenmoser et al., 2015). However, there is significant variation with subspecies and geographical range (Kaczensky et al., 2012). For example, the Balkan lynx (*L. l. balcanicus*) is classified as critically endangered with an estimated wild population of only 40–50 animals (Kaczensky et al., 2012, Melovski 2012) and the Ukrainian Eurasian lynx population is reportedly decreasing (Bashta & Dykyy, 2013; Shkvyria & Shevchenko, 2009).

Previous studies have investigated the causes of mortality in wild populations of Eurasian lynx and have shown by far the most common causes of mortality to be anthropogenic (54–97%) (Ryser‐Degiorgis, 2009). These causes include poaching, traffic collisions and hunting. Starvation, sarcoptic mange, and intraspecific killing were also recorded but were far less frequent (Andren et al., 2006; Ryser‐Degiorgis, 2009). In captivity, however, anthropogenic factors and starvation would be expected to be extremely rare, suggesting that a study of mortality in captive populations might bring to light prevalent disease processes masked by the abundance of human‐related mortality in wild populations. A previous study investigated the causes of morbidity and mortality in captive Iberian lynx and found iatrogenic vitamin D toxicosis (33%; 5/15) and intraspecific injury (20%; 3/15), often secondary to inter‐sibling aggression, to be the most prevalent causes of mortality in non‐neonatal individuals. Mortality in neonates (63%) was higher than in non‐neonates (37%) and was most commonly secondary to abortion, premature birth, still birth, and maternal neglect (Martinez, Manteca, & Pastor, 2013).

A comparison of the prevalence of cardiomyopathies in Eurasian lynx from Swiss populations and those from Swedish populations showed a higher prevalence in the Swiss sample. These Swiss populations originate from around 25 individuals reintroduced into the wild in the 1970's (now around 130 individuals in the Jura Mountains and Alps) and Ryser‐Degiorgis and Robert (2006) hypothesize that this pattern could be secondary to inbreeding depression following this “bottle‐neck” effect. Myocardial fibrosis and arteriosclerosis were the characteristic lesions detected at *post‐mortem* examination (PME). In addition to these cardiac lesions, congenital abnormalities of the genital organs, diaphragm, and skeleton were observed within the same Swiss populations (Ryser‐Degiorgis et al., 2004).

Prior to the establishment of the European studbook for Eurasian lynx in 2002, the level of inbreeding in captive European populations was reportedly “very high” (Versteege, 2006). It would, therefore, be reasonable to hypothesize that captive populations of Eurasian lynx could be predisposed to congenital abnormalities, including cardiomyopathy, as were observed in Swiss populations (Ryser‐Degiorgis & Robert, 2006).

The aim of this study is to assess the causes of mortality in captive Eurasian lynx. The objectives are as follows:

1. To identify the most common causes of mortality in Eurasian lynx in UK zoos.
2. To compare causes of mortality between age class, sex, subspecies, and inbreeding coefficient.
3. To compare ages of death between sex, subspecies, and inbreeding coefficient.
4. To make recommendations on areas in need of further research in the future.

## 2 MATERIALS AND METHODS

Using the European studbook (Versteege, 2015), we gathered data on all Eurasian lynx (hereby referred to as “lynx”) to have died in UK zoos between January 1, 2000 and November 1, 2015. This data included date and location of birth, the dam, and sire of each individual and, where available, information alluding to the cause of death. We contacted any existing institutions which had housed one or more Eurasian lynx at their time of death during the study period and requested PME reports. If PME had not been performed or had not been recorded adequately, we requested individual animal records. By analyzing these records, we attributed a cause of death where possible. If we could not reasonably ascertain a cause of death, it was recorded as “unknown.”

We categorized animals included in the study into groups depending on the age at death for statistical analysis. We classed any individuals which died prior to the normal age of weaning (4 months) (Kvaam, 1990; Breitenmoser, 1993) as “neonates.” “Juvenile” referred to lynx that survived beyond weaning but did not reach the average age of sexual maturity which is 24 months in females and 33 months in males (Krelekamp, 2004). We classed any lynx which died at reproductive age as “adult.” This corresponds to females between the ages of 24 months and 14 years, and males aged between 33 months and 17 years (Henriksen et al., 2005; Nowell & Jackson, 1996). “Geriatric” refers to any individuals which survived beyond reproductive age.

We categorized causes of death using a modified version of the World Health Organization's International Classification of Diseases (ICD‐10; Version 2016). We organized each cause of death into one of the following categories: infectious/parasitic, neoplastic, hematological, endocrine/nutritional/metabolic, neurological (including behavioral), ophthalmic, aural, circulatory, respiratory, digestive, skin/subcutaneous, musculoskeletal/connective tissue, genitourinary, diseases of pregnancy/parturition/puerperium (including abortion/stillbirth), congenital malformations/abnormalities, or injury/poisoning/external factors (e.g. iatrogenic death, trauma etc.). We added one extra category, “cull” (euthanasia of healthy animals as part of population management), to this system. We did not include animals which were euthanized on welfare grounds in this category and instead categorized these individuals according to the pathology leading to the decision to euthanize.

We categorized cases in which we could not reasonably identify a cause of death depending on the reason behind the ambiguity: “idiopathic” (adequate reports provided but cause of death not identified), “insufficient carcass quality” (autolyzed, eaten etc.), “institution abstinence” (institution did not respond or refused to supply data), or “insufficient data” (deficient records, lost records etc.). We then further categorized each cause of death as “euthanasia,” “non‐euthanasia,” or “unknown.”

With data from the European studbook (Versteege, 2015), we constructed pedigree charts and calculated inbreeding coefficients (COI) when sufficient data was available. We devised a novel classification system whereby individuals were categorized as “high COI” (>0.25; offspring of full‐sibling or parent‐offspring mating) or “low COI” (<0.25; any individuals with sufficient lineage data to calculate a theoretical maximum COI of less than 0.25). Although there is no widely recognized definition of a “high” COI, we selected this value as it facilitated calculation and identification of individuals with a theoretical maximum COI of less than 0.25 with limited heritage data available (i.e., any individuals with parents which were not full siblings or parent/offspring).

We calculated the mean age of death and used the standard error of the mean to describe variation (R version 3.2.2 [R Core Team 2015]). We then excluded “culled” individuals and “neonates” and recalculated the mean age at death to give a more accurate representation of natural lifespan in captivity, following completion of weaning. We then calculated mean age at death for each sex and subspecies, excluding “culled” individuals, to better represent any intrinsic variation between groups. We assessed any effects of sex and subspecies on age at death with a Welch's *t*‐test (R version 3.2.2 [R Core Team 2015]). We then calculated and compared the mean age of death for individuals of “high” and “low” COI. We included “culled” individuals in this calculation as they accounted for 100% of the “high” COI group.

We calculated the prevalence of the causes of death for the whole population and for each sex, age group, sub‐species, and COI. We drew comparisons and used chi‐square tests (R version 3.2.2 [R Core Team 2015]) to identify any meaningful variation. We included “culled” individuals in the age group comparison to illustrate the high prevalence of “neonatal culling,” and in the COI comparison. As with the age at death comparisons, we excluded culled individuals prior to comparisons of age and cause of death between subspecies and sex to better represent any intrinsic variation. We also used chi‐square tests (R version 3.2.2 [R Core Team 2015]) to identify any significant disparity between the number of males and females which deceased within the study period.

## 3 RESULTS

The study sample comprised a total of 52 individuals from 18 UK institutions. Of these 52 animals, 48% (25/52) were male and 52% (27/52) were female, showing no significant sex bias (*p* = 0.78).

**Figure 1:**
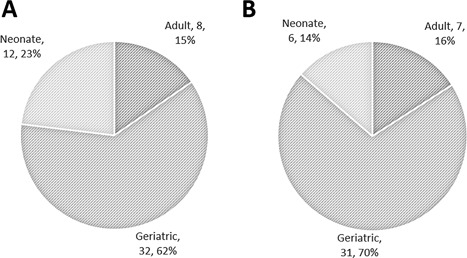
Title: Sample distribution between age groups in UK captive Eurasian lynx (*Lynx lynx*). Footnote: (A) All individuals to die within study period (*n* = 52); (B) as for A with culled individuals excluded (*n* = 44). Absolute numbers and percentages provided

The majority (62%; 32/52) of deaths during the study period were in “geriatric” animals. Twenty‐three percent (12/52) of individuals to die during the study period were “neonates” and the remaining 15% (8/52) were “adult” (Figure 1). No “juvenile” lynx died during the study period.

**Figure 2:**
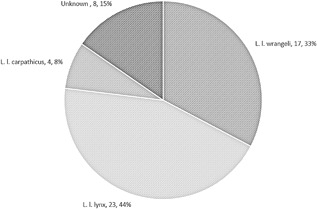
Title: Sample distribution between UK captive Eurasian lynx (*Lynx lynx*) subspecies. Absolute numbers and percentages provided (*n* = 52)

The most common sub‐species included in the study was the Northern lynx, making up 44% (23/52) of the total sample. The Siberian lynx (33%; 17/52) was the second most common with the Carpathian lynx (8%; 4/52) and lynx of unknown subspecies (15%; 8/52) present in smaller numbers (Figure 2).

### 3.1 Age at death

The average age at death during the study period was 14.27 ± 1.16 years. Eight individuals (15%; 8/52) were “culled” as part of captive population management. With these individuals excluded the mean age at death becomes 16.24 ± 0.98 years. Further excluding six “neonate” individuals, the mean age at death becomes 18.81 ± 0.42 years.

**Figure 3:**
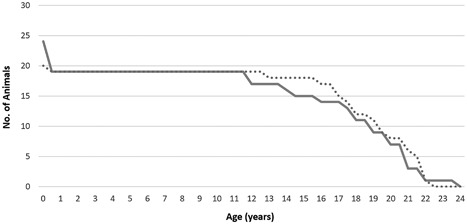
Title: Number of individuals of each sex to reach respective age in UK Captive Eurasian lynx (*Lynx lynx*). Footnote: Culled individuals are excluded (*n* = 44). Dotted: males; solid: females

Excluding “culled” individuals, male lynx reached a mean age of 18.32 ± 0.69 years and females reached 14.51 ± 1.13 years, showing no significant variation (*p* = 0.065). Figure 3 shows the ages reached by animals during the study period with “culls” excluded. Females exhibited higher levels of “neonatal” mortality (21%; 5/24) than males (5%; 1/20) (*p* = 0.13). Also excluding neonates, males had a mean age at death of 19.29 ± 0.36 years compared to 18.33 ± 0.47 years in females (*p* = 0.09). The oldest individual included in the study was a female Northern lynx which reached an age of 23 years and 2 months.

Excluding “culls,” Northern lynx (*n* = 23) had a mean age at death of 18.43 ± 0.47 years, Siberian lynx (*n* = 11) of 15.66 ± 1.14 years, and lynx of unknown subspecies (*n* = 8) of 19.43 ± 0.26 years (no significant variation; *p* > 0.05). Carpathian lynx (*n* = 4) demonstrated a significantly lower average age of mortality, when compared to rest of the sample, at 0.16 ± 0.01 months (*p *< 0.0001).

It was possible to calculate a COI for only 20/52 lynx (38%). Of these, 65% (13/20) had a “low” COI (<0.25) and 35% (7/20) had a “high” COI (>0.25). All seven individuals with a “high” COI were “culled” therefore any effects of COI on natural age at death cannot be meaningfully analyzed.

### 3.2 Cause of death

Whether death occurred naturally or due to euthanasia was reported in 85% of cases (44/52). 70% (31/44) of these individuals were euthanized with the remaining 30% (13/44) found dead.

Of the 52 individuals included in the study, we were able to ascertain a cause of death for 73% (38/52). For the remaining 27% (14/52) a cause of death could not be identified due to “insufficient records” (15%; 8/52), “institution abstinence” (10%; 5/52), or “insufficient carcass quality” (2%; 1/52). The quality and quantity of data provided was varied: 61% (23/38) of causes of death were identified via interpretation of comprehensive PME reports, 24% (9/38) were gleaned from data recorded in the European studbook and we identified the remaining 16% (6/38) using individual animal records.

### 3.3 Effect of age group on cause of death

The causes of death by age group can be seen in Table 1. Within the total sample, “cull” (8/38; 21%) was the leading cause of death followed by “neoplastic” disease (6/38; 16%). “Circulatory,” “genitourinary,” and “neurological” (4/38; 11%) were the remaining disease types to constitute more than 10% of the total sample. The high incidence of culling as a cause of death was largely observed in the “neonate” category where it accounted for 55% (6/11) of all deaths. In the “adult” age group, “circulatory” disease was the most prevalent cause of death, accounting for 25% (2/8) of the sample. In “geriatric” animals, “neoplastic” disease was the cause of death in 26% (5/19) of individuals with “genitourinary” disease (4/19; 21%) and “neurological” disease (3/19; 16%) also prevalent.

**Table 1:** Causes of death in Eurasian lynx (*Lynx lynx*) for which a cause of death could be identified (*n* = 38) by age group

Cause of Death	Neonate	Adult	Geriatric	Total
Circulatory	‐	2 (25%)	2 (11%)	4 (11%)
Cull	6 (55%)	1 (13%)	1 (5%)	8 (21%)
Diseases of pregnancy/parturition/puerperium	2 (18%)	1 (13%)	‐	3 (8%)
Genitourinary	‐	‐	4 (21%)	4 (11%)
Infectious/parasitic	1 (9%)	‐	‐	1 (3%)
Injury/poisoning/external factors	1 (9%)	1 (13%)	‐	2 (5%)
Musculoskeletal/connective tissue	‐	1 (13%)	2 (11%)	3 (8%)
Neoplastic	‐	1 (13%)	5 (26%)	6 (16%)
Neurological	‐	1 (13%)	3 (16%)	4 (11%)
Respiratory	1 (9%)	‐	2 (11%)	3 (8%)
Total	11	8	19	38

Absolute numbers and percentages provided

### 3.4 Effect of sex on cause of death

The causes of death within each sex can be seen in Table 2. Following exclusion of “culls,” the most prevalent cause of death within the male sample was “circulatory” disease (27%; 3/11) followed by “respiratory” disease (18%; 2/11) and musculoskeletal/connective tissue disease (18%; 2/11). In females, neoplastic disease (26%; 5/19) was the leading cause of death, followed by neurological (16%; 3/22) and genitourinary disease (16%; 3/22).

**Table 2:** Causes of death in Eurasian lynx (*Lynx lynx*), excluding culled animals, for which a cause of death could be identified, (*n* = 30) by sex

Cause of Death	Female	Male	Total
Circulatory	1 (5%)	3 (27%)	4 (13%)
Diseases of pregnancy/parturition/puerperium	2 (11%)	1 (9%)	3 (10%)
Genitourinary	3 (16%)	1 (9%)	4 (13%)
Infectious/parasitic	1 (5%)	‐	1 (3%)
Injury/poisoning/external factors	2 (11%)	‐	2 (7%)
Musculoskeletal/connective tissue	1 (5%)	2 (18%)	3 (10%)
Neoplastic	5 (26%)	1 (9%)	6 (20%)
Neurological	3 (16%)	1 (9%)	4 (13%)
Respiratory	1 (5%)	2 (18%)	3 (10%)
Total	19	11	30

Absolute numbers and percentages provided

### 3.5 Effect of subspecies on cause of death

As reported in Table 3, the leading cause of death, excluding “culls,” in Northern lynx was neoplastic disease (47%; 6/19) followed by “neurological” and “musculoskeletal/connective tissue” disease (both 16%; 3/19). Siberian lynx died most frequently due to “diseases of pregnancy/parturition/puerperium” (38%; 3/8) and circulatory disease (25%; 2/8). The causes of death in Carpathian lynx were “infectious/parasitic” diseases, “injury/poisoning/external factors” and “respiratory” disease with each accounting for 33% (1/3) of deaths.

**Table 3:** Causes of death in Eurasian lynx (*Lynx lynx*), excluding culled animals, for which a cause of death could be identified, (*n* = 30) by subspecies

Cause of Death	*L. l. lynx*	*L. l. wrangeli*	*L. l. carpathicus*	Total
Circulatory	2 (11%)	2 (25%)	‐	4 (13%)
Diseases of pregnancy/parturition/puerperium	‐	3 (38%)	‐	3 (10%)
Genitourinary	3 (16%)	1 (13%)	‐	4 (13%)
Infectious/parasitic	‐	‐	1 (33%)	1 (3%)
Injury/poisoning/external factors	1 (8%)	‐	1 (33%)	2 (7%)
Musculoskeletal/connective tissue	3 (16%)	‐	‐	3 (10%)
Neoplastic	6 (47%)	‐	‐	6 (20%)
Neurological	3 (16%)	1 (13%)	‐	4 (13%)
Respiratory	1 (8%)	1 (13%)	1 (33%)	3 (10%)
Total	19	8	3	30

Absolute numbers and percentages provided

#### 3.5.1 Culling

Six “neonates,” one “adult,” and one “geriatric” were culled during the study period. 88% (7/8) of these individuals had a “high” COI (<0.25).

#### 3.5.2 Neoplasia

Of the six cases of fatal neoplasia during the study period, five were histopathologically diagnosed as carcinomas. Three were squamous cell carcinomas (SCC) with sebaceous carcinoma and adrenocortical carcinoma each diagnosed in a single case. All six cases of fatal neoplasia during the study period affected the Northern lynx (*p* = 0.11) and were unsurprisingly seen predominantly in the “geriatric” age group when compared to the rest of the sample (83%; 5/6) (*p* = 0.08).

#### 3.5.3 Neurological

Idiopathic epilepsy was the diagnosed cause of death in all four individuals to die from neurological disease during the study period. Three of the four individuals were within the “geriatric” age group, with no significant variation in prevalence of neurological disease among age groups (*p* = 0.70).

#### 3.5.4 Circulatory

Cardiomyopathy accounted for three of the four cases of circulatory disease. Affected animals belonged to the “adult” (2/4) and “geriatric” (2/4) age groups, with the youngest individual affected a 12 year and 9‐month‐old male Northern lynx. Circulatory disease was not significantly more prevalent within the “adult” group when compared to the rest of the sample (*p* = 0.13). Males (27%; 3/11) expressed a higher prevalence of fatal circulatory disease than females (5%; 1/19) during the study period (*p* = 0.25).

#### 3.5.5 Genitourinary

Protein‐losing nephropathy was a feature of all individuals to die due to genitourinary disease during the sample period. Three of the four individuals displayed chronic interstitial lymphoplasmacytic nephritis and glomerular sclerosis at PME, suggestive of chronic renal disease. Fatal genitourinary disease was observed only in “geriatric” animals during the sample period, with no significant variation in prevalence among age groups (*p* = 0.42).

#### 3.5.6 Pregnancy/parturition/puerperium

One “adult” female and two “neonates” died during parturition, however, no further explanation of the pathology causing the deaths was available.

#### 3.5.7 Respiratory

Three individuals suffered fatal respiratory disease during the study period. Sub‐acute to chronic alveolar oedema was detected at PME in two “geriatric” animals and one “neonate” died as a result of non‐infectious atelectasis.

#### 3.5.8 Musculoskeletal/connective tissue

One “adult” and two “geriatric” animals were euthanased on welfare grounds as a result of severe alkylosing spondylosis and degenerative joint disease, respectively.

#### 3.5.9 Other

Of the remaining three individuals for which causes of death were identified, two were “neonates.” One died as a result of haemorrhagic gastroenteritis due to primary bacterial infection of *Escherichia coli*. The other died as a result of cranial trauma. One “adult” lynx died as a result of severe soft‐tissue damage following intra‐specific aggression.

## 4 DISCUSSION

### 4.1 Age at death

Following exclusion of neonates and “culled” animals, the average age of death in this study was 18.81 years with the oldest animal reaching an age of 23 years and 2 months. This is consistent with existing data on captive lynx mortality which states captive individuals can live up to 25 years (von Arx, Breitenmoser‐Würsten, Zimmerman, & Breitenmoser, 2001). Unsurprisingly, wild lynx have not been reported to reach similar ages, with the oldest wild lynx reportedly dying at an age of 17 years (Breitenmoser,^1993^; Kvaam, 1990).

Martinez et al. (2013) identified high levels of mortality in captive Iberian lynx of less than one week of age, with 63% (25/40) of deaths occurring within this age group. However, animals aged 1 week or less accounted for only 11% of deaths (5/44) in Eurasian lynx in this study.

### 4.2 Cause of death

The most common cause of death within the study period was “culling,” accounting for eight of the 38 (21%) deaths of known cause. Of the eight culled individuals, seven (six “neonate”; one “adult”) were categorized as having a “high” COI. One was the progeny of parent‐offspring breeding and six were the offspring of a single sire following inter‐sibling breeding. Unfortunately, no PME reports were provided for six of the seven animals with a known “high” COI in this study, severely impairing comparison with observations in wild Swiss populations reported by Ryser‐Degiorgis et al. (2004). In the one individual with a “high” COI, for which a PME report was provided, no congenital malformations were detected. In fact, “Congenital malformations/abnormalities” were not recorded as the cause of death for any individuals during the study period. The hypothesis that captive lynx populations are undergoing similar changes to those observed in wild Swiss populations can be neither accepted nor rejected due to the shortage of data.

Cardiomyopathy accounted for three of the four recorded cases of “circulatory” disease. In wild Swiss lynx, cardiomyopathy was present at PME in 95% of adults, with interstitial myocardial fibrosis and arterioscelerosis the most common findings (Ryser‐Degiorgis & Robert, 2006). In this study, no individual displayed both of these lesions, however, two individuals exhibited mild arteriosclerosis at PME and moderate myocardial fibrosis was detected in one animal. It is unclear if this is a manifestation of the same syndrome described by Ryser‐Degiorgis and Robert (2006) in wild Swiss lynx or unrelated degenerative change. Males exhibited a higher prevalence of cardiomyopathy than females in wild Swiss populations (Ryser‐Degiorgis & Robert, 2006) and this study's findings were consistent with this; 27% (3/11) of deaths of known cause in males were due to “circulatory” disease compared to just 5% (1/19) in females (*p* = 0.25).

There were three cases of fatal SCC within the study period; two “geriatric” individuals and one “adult.” One was nasal in origin, one gingival, and one cutaneuous (located at the base of the pinna). Two types of neoplasia have been previously reported in Eurasian lynx; a multi‐hormonal endocrine pancreatic tumor (Kirchhof & Geiss, 1995) and a benign giant cell tumor of tendon sheaths of the forelimb (Malatesta et al., 2005). Gingival SCC has been reported in Canada lynx (Gunson, 1978) and other non‐domestic felids (Kesdangsakonwut, Sanannu, Rungsipipat, & Banlunar, 2014).

Seizures were reported as the cause of death in three “geriatric” animals and one “adult.” In each of these cases, PME revealed no etiology for seizures (e.g., hepatic encephalopathy, structural brain lesions). Suspected idiopathic epilepsy has been reported in captive Iberian lynx, reportedly affecting 4% (4/96) of individuals (Martinez et al., 2013).

Martinez et al. (2013) reported genitourinary disease as the cause of death for 33% (5/15) of captive Iberian lynx of over one week of age compared to just 13% (4/30) of Eurasian lynx in this study. Every case of genitourinary disease in the Iberian lynx study, however, was secondary to iatrogenic vitamin D toxicosis, which was not detected at all within the UK Eurasian lynx population during the study period. Fatal genitourinary disease in this study was seen only in “geriatric” individuals with chronic interstitial lyphoplasmacytic nephritis and glomerular scelrosis detected in three of the four cases indicating degenerative renal disease. PME was not performed for one individual, although routine biochemistry and urinalysis revealed severe azotaemia and proteinuria shortly prior to death, highly indicative of renal failure in felids (Paepe & Daminet, 2013). Glomerulosclerosis (Bolton & Munson, 1999) and interstitial lymphoplasmacytic nephritis (Newkirk, Newman, White, Rohrbach, & Ramsay, 2011) have been described in other non‐domestic felids.

Previous studies have reported high levels of inter‐sibling aggression in captive Eurasian lynx (Naidenko & Antonevich, 2009; Sokolov, Naidenko, & Serbenyuk, 1994), making it one of very few mammalian species to display this behavior (Fraser, 1990; Frank, Glickman, & Light, 1991; Ovsyannikov, 1993). The aforementioned study of captive Iberian lynx mortality reported 10% (4/40) of deaths during the study period as a result of “intra‐specific trauma,” with 76% of all observed aggressions between siblings (Martinez et al., 2013), suggesting that this behavior could be shared by Iberian lynx. In this study, however, only one individual (3%) died as a result of intraspecific aggression; an “adult” female Northern lynx which was euthanized following severe skin lacerations inflicted by another unrelated female.

Studies in wild Eurasian lynx have reported the most common causes of mortality to be anthropogenic with only 3–52% of deaths due to disease (Ryser‐Degiorgis, 2009). However, as mortality studies in wild populations that do not utilize radio‐collars to locate deceased animals rely solely on dead animals found by chance, it is possible that anthropogenic mortality is over‐represented. However, even studies using radio‐collars have found proportions of mortality from poaching as high as 46% in some Swedish populations (Andren et al., 2006).

In a study of radio‐collared Swiss lynx, 40% of individuals exhibited fatal “infectious/parasitic” disease with bacterial bronchopneumonia, toxocariasis, sarcoptic and notoedric mange being the most common (Schmidt‐Posthaus, Breitenmoser‐Würsten, Posthaus, Bacciarini, & Breitenmoser, 2002). This disease category only accounted for one death in UK zoos during the study period, however, this is perhaps unsurprising as captive animals are monitored with veterinary care provided when necessary.

### 4.3 Limitations

The primary limitations of this study are the small sample size (*n* = 52) and the varied quality and quantity of data provided. Of the 52 individuals in the study, data relating to the cause of death was available for just 73%. This was largely due to “insufficient records” (often records had been lost prior to digitalization of record‐keeping with ZIMS) and “institution abstinence” whereby institutions did not respond to the request for data or refused to provide it. For 40% of animals, comprehensive PME reports were not provided and we ascertained a cause of death via interpretation of individual animal records (often including a brief summary of PME findings) or the European studbook (Versteege, 2015).

PME reports were not available for six (86%) of the seven individuals with a “high” COI. Had PME data been provided for these animals, we may have detected congenital malformations similar to those described by Ryser‐Degiorgis et al. (2004) and could have compared these in more detail to those found in reintroduced Swiss lynx populations.

## 5 CONCLUSIONS

1. The general mortality trends identified in this study can be used to aid diagnosis and improve preventative health care strategies in captive Eurasian lynx.
2. The high prevalence of suspected idiopathic epilepsy (4/38; 11%) and SCC (three of six cases of neoplastic disease) warrants further investigation. SCC has not been previously reported in Eurasian lynx and is a malignant and locally invasive neoplasm with a generally poor prognosis in domestic animals (Webb, Burns, Brown, LeRoy, & Kosarek, 2009).
3. This study reasserts the advice of the studbook coordinator; future breeding should be conducted pending consultation of the studbook with all other breeding ceased (Versteege, 2006). While culling is a legitimate captive population management strategy (EAZA, 2015), seven of the eight reported culls during the study period occurred in lynx with a “high” COI and took place prior to 2005 with all “neonatal” culls (6/8) occurring prior to the establishment of the European studbook in 2002. Inbreeding poses a danger to the whole captive population by reducing genetic variation so should be addressed stringently.
4. Due to limited available data for individuals with a “high” COI, it was not possible to compare the potential effects of inbreeding to the abnormalities reported in wild Swiss populations by Ryser‐Degiorgis et al. (2004). A future morbidity and mortality study of individuals from bloodlines of known high levels of inbreeding would allow superior analysis of the effects of inbreeding. A larger sample size could be achieved by including all captive Eurasian lynx in Europe with the implementation of a standardized PME protocol.
5. Improved PME reporting and record keeping would be enormously beneficial to future research. The use of computerized systems such as ZIMS would better facilitate this and minimize the number of “insufficient records,” which accounted for 57% (8/14) of cases for which the cause of death could not be identified.

## CONFLICTS OF INTEREST

The authors have no conflict of interest to declare in relation to this work.
